# Nanotechnology-driven STING regulation for on-demand therapy

**DOI:** 10.1016/j.apsb.2026.02.005

**Published:** 2026-02-10

**Authors:** Qianwen Mu, Qihang Huang, Haolan Deng, Gang Liu, Chao Liu

**Affiliations:** a State Key Laboratory of Vaccines for Infectious Diseases, Xiang An Biomedicine Laboratory, and Fujian Provincial Key Laboratory of Innovative Drug Target Research, School of Pharmaceutical Sciences, Xiamen University, Xiamen 361102, China; b State Key Laboratory of Vaccines for Infectious Diseases, Xiang An Biomedicine Laboratory, Fujian Engineering Research Center of Molecular Theranostic Technology, School of Public Health, Xiamen University, Xiamen 361102, China; c State Key Laboratory of Cellular Stress Biology, Innovation Center for Cell Biology, School of Life Sciences, Xiamen University, Xiamen 361102, China; d Shenzhen Research Institute of Xiamen University, Shenzhen 518000, China

**Keywords:** cGAS–STING pathway, Nanotechnology, Nanomedicine, Immunotherapy, Cancer, Inflammation, Autoimmunity, Infection

## Abstract

The combination of cyclic GMP–AMP synthase (cGAS)-stimulator of interferon genes (STING) signaling pathway modulation with nanotechnology offers a promising strategy for the development of more effective and less toxic therapies. This review summarizes the latest clinical progress of STING agonists and inhibitors, with a particular focus on the role of nanomaterials in regulating the cGAS–STING pathway across a range of diseases. In oncology, STING activation enhances anti-tumor immunity by stimulating immune cells, while nanocarriers improve the stability and targeting precision of STING agonists, facilitating synergistic effects with other immunotherapies. In inflammatory and autoimmune diseases, regulating STING activation helps alleviate the production of excessive pro-inflammatory cytokines, restore immune homeostasis, and prevent tissue damage. Nanomaterials, such as cell-derived membranes, further enhance targeted delivery and biocompatibility, addressing key limitations of existing treatment strategies. What distinguishes this review is an in-depth analysis of the current clinical progress of STING agonists and inhibitors, providing a comprehensive overview of both ongoing clinical trials and preclinical advancements. We also critically evaluate the specific challenges encountered in translating STING nanomaterials into clinical practice. These challenges present significant barriers to the widespread application of STING-based therapies, underscoring the need for further optimization to realize their full potential.

## Graphical abstract

This review systematically explores the therapeutic potential of nanotechnology-driven STING targeting for various diseases.

**Figure:**
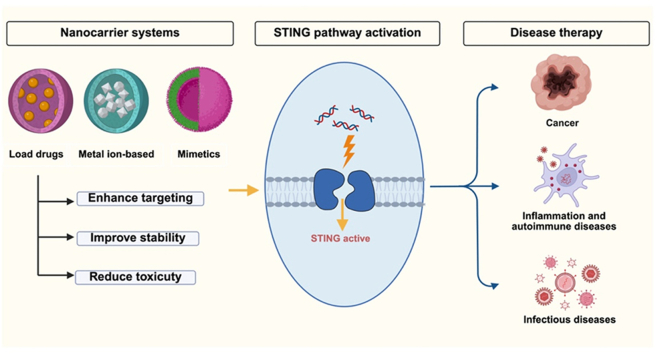

## 1 Introduction

The cyclic GMP–AMP synthase (cGAS)-stimulator of interferon genes (STING) signaling pathway is a critical component of the innate immune system, governing pathogen recognition, immune activation, and inflammation regulation^1^^,^^2^. In recent years, STING agonists have emerged as a novel class of immune modulators with therapeutic potential across multiple disease domains^3^^,^^4^. In cancer immunotherapy, STING agonists promote the maturation of dendritic cells (DCs), enhance tumor antigen presentation, and induce type I interferon production, thereby activating CD8^+^ T cell-mediated anti-tumor immune responses and reprogramming the “cold tumor” microenvironment^5^, ^6^, ^7^, ^8^. In autoimmune diseases, sustained and aberrant cGAS–STING pathway activation is recognized as a key pathogenic mechanism^1^^,^^9^. Research on STING not only deepens the understanding of disease mechanisms but also underscores the need to carefully evaluate potential side effects of STING agonists in clinical applications. In infectious diseases, STING agonists enhance the host's innate immune defenses against DNA viruses and certain bacteria, improve vaccine immunogenicity, and show promise as adjuvants for infection and cancer vaccines^10^, ^11^, ^12^.

**Figure 1:**
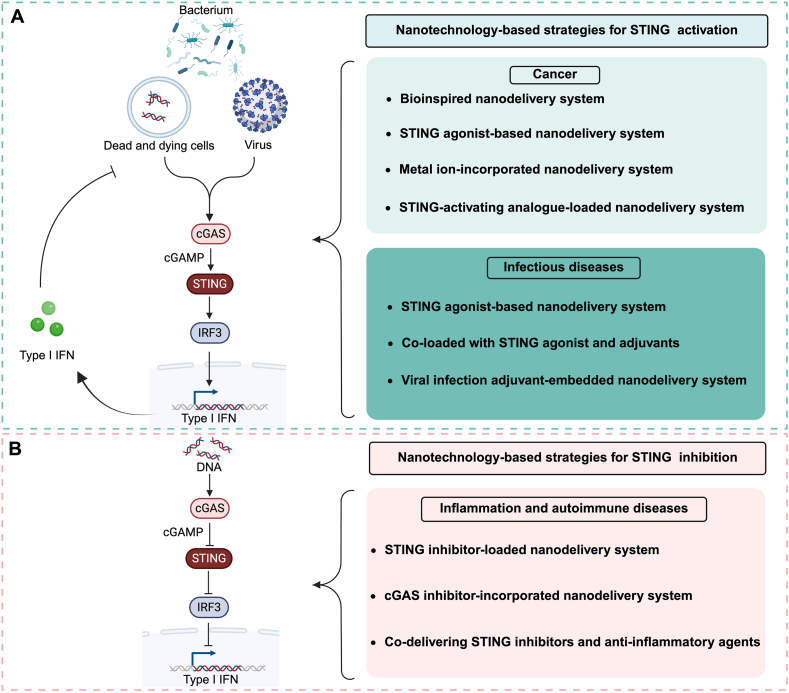
Nanotechnology-driven STING regulation for diseases. (A) Nanotechnology-based strategies for cGAS–STING pathway activation in cancer and infectious diseases. STING signaling can be potentiated through nanotechnology-driven approaches, such as STING agonist-loaded nanodelivery systems or targeted STING-activating nanoformulations to enhance immunostimulatory efficacy. These strategies aim to amplify cytosolic DNA sensing and downstream interferon responses, promoting antitumor immunity or antiviral defense. (B) Nanotechnology-based cGAS–STING pathway inhibition. For autoimmune and inflammatory disorders, excessive STING activation can be suppressed *via* nanoparticle-based delivery of STING inhibitors, cGAS-targeting therapeutics, or combinatorial nanoformulations co-encapsulating anti-inflammatory agents. These approaches mitigate pathological inflammation by modulating aberrant innate immune signaling, thereby offering targeted intervention for conditions driven by STING hyperactivation. Created by BioRender.com.

As a crucial link between innate and adaptive immunity, STING agonists and inhibitors exhibit multiple mechanisms of action and considerable clinical translation potential^13^, ^14^, ^15^. However, the risks of inflammatory toxicity and immune dysregulation in various disease models necessitate further investigation and careful management^16^. This review provides a comprehensive analysis of the immunoregulatory mechanisms of the cGAS–STING pathway in cancer, inflammation, autoimmune diseases, and infections^17^. What sets this review apart from other similar articles is the emphasis on the dual role of nanomaterials in both positively and negatively modulating the cGAS–STING signaling pathway, and how these effects are disease-dependent (Fig. 1). We also provide an in-depth exploration of the challenges encountered in the clinical translation of STING-based therapies, specifically addressing issues such as stability, toxicity, and immune-related adverse events.

In addition, we highlight emerging strategies for STING agonist therapy utilizing nanodelivery systems (Fig. 1), which have the potential to enable precise delivery and targeted modulation. This approach aims to enhance therapeutic efficacy while minimizing off-target effects, thus offering a fresh perspective and promising prospects for the precise immunotherapy of STING-related diseases. Through this unique focus, we provide insights into the broader implications of STING modulation by nanomaterials and discuss the specific hurdles in translating these therapies into clinical practice.

## 2 Regulation of the cGAS–STING signaling pathway in diseases

### 2.1 Regulation of the cGAS**–**STING pathway in cancer

The cGAS–STING signaling pathway has garnered increasing attention in immunotherapy, particularly as a promising therapeutic target for cancer^2^^,^^8^^,^^18^, ^19^, ^20^. Activation of this pathway enhances anti-tumor immune responses by promoting malignant cell clearance, offering new opportunities for cancer immunotherapy beyond its role in innate immune defense. Concurrently, cGAS and STING have become focal points for developing novel immune-based therapeutic strategies^21^, ^22^, ^23^, ^24^, ^25^.

The anti-tumor effects of the cGAS–STING pathway are mediated through both cell-intrinsic and cell-extrinsic mechanisms. Intrinsically, oncogene-induced senescence triggers the secretion of senescence-associated secretory phenotypes, inhibiting tumor cell proliferation^26^^,^^27^. STING activation within tumor cells further induces apoptosis to suppress tumor growth. Extrinsically, STING activation drives type I interferons (IFN-I) transcription, facilitating crosstalk between tumor cells and immune components to orchestrate anti-tumor immunity. DCs play a key role by capturing tumor-associated antigens and initiating cytotoxic CD8^+^ T cell responses^28^^,^^29^. Macrophages support the anti-tumor microenvironment by secreting tumor necrosis factor-alpha (TNF-*α*) and expressing chemokines (*e*.*g*., C–X–C motif chemokine ligand 10 (CXCL10) and C–X–C motif chemokine ligand 11(CXCL11)) and nitric oxide synthase 2 (NOS2)^30^, ^31^, ^32^, ^33^. Stromal cells contribute to anti-angiogenic effects *via* interferon-beta (IFN-*β*) production^18^^,^^34^.

During tumorigenesis, genomic DNA alterations trigger DNA damage response (DDR), chromosome instability (CIN), and micronuclei formation. Genomic instability transiently activates the cGAS–STING pathway, recruiting CD8^+^ T cells and natural killer (NK) cells to eliminate cancer cells. Activated cGAS–STING induces interferon production, critical for immune surveillance, DC activation, and CD8^+^ T cell-mediated anti-tumor immune responses^35^^,^^36^. DDR pathways reflect the necessity of sensing and repairing various forms of DNA damage. Deficiencies in DDR can lead to increased DNA damage, the accumulation of cytosolic DNA, and the activation of the cGAS–STING pathway^37^, ^38^, ^39^, ^40^. Genes encoding DDR components are frequently mutated in cancer cells, impairing DNA repair and contributing to CIN. CIN results from errors in chromosome segregation during mitosis, leading to aneuploidy and micronucleus formation^41^, ^42^, ^43^, ^44^. Micronuclei are membrane-bound vesicles containing DNA, although they originate from the nuclear envelope, they are unstable. In cancers with a high CIN phenotype (CIN^hi^), micronuclei rupture, releasing cytosolic DNA, which can persist and potentially activate the cGAS–STING pathway, thus eliciting an anti-tumor immune response^45^, ^46^, ^47^, ^48^.

**Figure 2:**
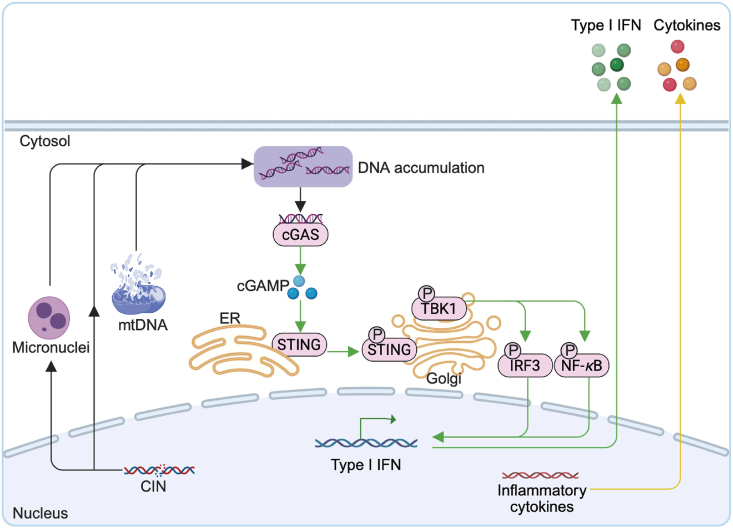
STING regulation in cancer. During tumorigenesis, genomic instability caused by CIN and DDR leads to the formation of micronuclei and the release of mitochondrial DNA (mtDNA), resulting in the accumulation of cytosolic DNA. The cGAS senses this cytosolic DNA and synthesizes the second messenger cGAMP, which activates STING located on the endoplasmic reticulum (ER). STING then translocates to the Golgi apparatus, where it recruits and activates the kinase TANK-binding kinase 1 (TBK1), leading to the phosphorylation of downstream transcription factors such as interferon regulatory factor 3 (IRF3) and nuclear factor kappa-light-chain-enhancer of activated B cells (NF-*κ*B). This activation induces the expression of IFN-I and inflammatory cytokines, which exert anti-tumor effects through both cell-intrinsic and cell-extrinsic mechanisms. These include enhancing DCs' function, promoting the activation of CD8^+^ T cells, and shaping an immune microenvironment favorable for tumor clearance. Created by BioRender.com.

Although significant progress has been made in understanding the role of the cGAS–STING axis in tumor immunity in recent years, many unresolved questions remain regarding how cGAS-mediated DNA sensing mechanisms regulate STING activation within cancer cells. The activation modalities and regulatory mechanisms of STING signaling in different tumor microenvironments, as well as its complex interactions with DNA repair pathways, require further in-depth investigation. The mechanisms of STING activation and its regulatory network in tumors are illustrated in Fig. 2.

### 2.2 Regulation of the cGAS**–**STING in inflammatory and autoimmune diseases

As a key pattern recognition and effector pathway in the innate immune system, the cGAS–STING signaling pathway is abnormally activated when pathogenic DNA or erroneous self-DNA is detected, leading to excessive production of IFN-I and the onset of inflammatory diseases^27^^,^^49^, ^50^, ^51^. Numerous studies have demonstrated a strong association between dysregulation of the cGAS–STING pathway and inflammation. Mutations that disrupt self-DNA metabolism, such as those in the DNA exonuclease TREX1, can lead to cGAS–STING activation and trigger inflammatory diseases^52^, ^53^, ^54^.

Autoimmune diseases primarily arise from an imbalance between the innate and adaptive immune systems, causing the body to fail in distinguishing between self and foreign antigens^55^, ^56^, ^57^, ^58^. Overactivation of the cGAS–STING pathway, resulting in the excessive release of IFN-I, is implicated in the pathogenesis of several autoimmune disorders, including inflammatory bowel disease (IBD), COPA syndrome, and systemic lupus erythematosus (SLE)^59^, ^60^, ^61^, ^62^.

IBD is a chronic inflammatory condition of the gut triggered by multiple factors. Recent studies have highlighted the frequent occurrence of abnormal cGAS–STING pathway activation in IBD patients, which is closely linked to intestinal immune dysregulation, chronic inflammation, and disruption of the intestinal barrier. In IBD, intestinal epithelial cells, immune cells (such as DCs and macrophages), and other gut-associated immune cells respond to DNA damage caused by gut microbiota or host cells *via* the cGAS–STING pathway. This overactivated immune response may lead to excessive production of IFN-I, which further exacerbates local intestinal inflammation. The activation of STING not only promotes the aggregation and activation of immune cells but may also impair the barrier function of intestinal epithelial cells, resulting in the breakdown of the gut barrier and reinforcing the inflammatory cycle^63^^,^^64^. SLE is a chronic autoimmune disease that can affect multiple organs and is characterized by systemic inflammation, the production of autoantibodies, and the deposition of immune complexes. The cGAS–STING pathway is considered a driving factor in SLE pathogenesis, with elevated levels of IFN-I and pro-inflammatory cytokines observed in the serum of SLE patients^65^.

Inhibiting the cGAS–STING pathway offers significant therapeutic potential in the treatment of inflammatory and autoimmune diseases^55^^,^^66^. As excessive STING activation leads to chronic inflammation and immune dysregulation, particularly through the overproduction of IFN-I, STING inhibition could help to restore immune balance. In diseases like IBD and SLE, where STING plays a central role in driving inflammation, targeting this pathway may reduce inflammation, prevent tissue damage, and improve patient outcomes^67^. Moreover, STING inhibition could offer a more targeted approach compared to general immune suppression, minimizing the risk of infections and other immune-related side effects while effectively managing disease activity^68^. Thus, modulating STING activity represents a promising strategy to treat immune-mediated conditions without compromising the body's defense against pathogens.

### 2.3 Regulation of the cGAS-STING pathway in infectious diseases

**Figure 3:**
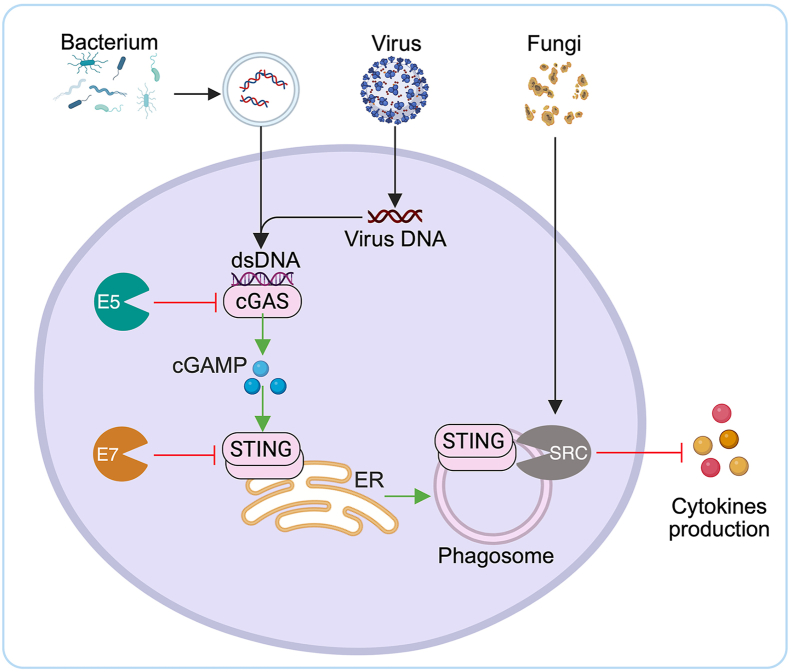
Regulation of cGAS-STING signaling pathways in infectious diseases. The cGAS can sense bacteria, DNA viruses, RNA viruses, and fungi. Upon detecting cytosolic double-stranded DNA (dsDNA) of pathogenic origin, such as bacterial DNA, viral genomes, or mitochondrial DNA released during infection, cGAS synthesizes the second messenger cGAMP, which activates STING on the ER. Activated STING translocates to the Golgi or phagosome compartments, where it initiates downstream signaling to induce IFN-I and inflammatory cytokines. However, certain viruses have evolved mechanisms to evade this immune detection: for example, vaccinia virus protein E5 inhibits cGAS activity, while HPV E7 inhibits STING activation. Additionally, fungal components can also engage STING signaling *via* phagosomal SRC kinase pathways, contributing to cytokine production. This complex regulation underscores the cGAS–STING pathway's central role in orchestrating innate immune responses against diverse microbial threats. Created by BioRender.com.

A critical function of the cGAS–STING signaling axis lies in host defense against microbial infections, including DNA viruses, RNA viruses, bacteria, and fungi. Upon sensing pathogen-derived nucleic acids, this pathway elicits a robust IFN-I response, thereby limiting pathogen replication and dissemination (Fig. 3). However, numerous viruses have evolved immune evasion mechanisms to counteract this pathway. For instance, viral effector proteins such as ORF52 of Kaposi's sarcoma-associated herpesvirus, E5 of vaccinia virus, and VP22 and UL37 of herpes simplex virus 1 can suppress cGAS activity^69^, ^70^, ^71^. Likewise, the E7 protein of human papillomavirus and the SRC kinase of fungi can inhibit STING activation^9^^,^^72^. In addition to DNA viruses, certain RNA viruses, such as enterovirus A71 (EV-A71), have also been shown to be restricted by cGAS–STING signaling^11^. Although the precise antiviral mechanisms by which cGAS–STING senses viral DNA remain incompletely understood, one proposed model suggests that viral infections induce the release of mtDNA, which subsequently activates cGAS and promotes innate antiviral responses^12^.

Several RNA viruses, including severe acute respiratory syndrome coronavirus 2 (SARS-CoV-2) and human immunodeficiency virus (HIV), are also capable of activating the cGAS–STING pathway^73^, ^74^, ^75^, ^76^. SARS-CoV-2 enters host cells *via* membrane fusion, mediated by the interaction of its spike protein with the angiotensin-converting enzyme 2 receptor. This process, along with virus-induced syncytia formation and cell–cell fusion, leads to cytosolic accumulation of genomic DNA, thereby activating cGAS–STING signaling^77^, ^78^, ^79^, ^80^, ^81^. In this context, STING undergoes phosphorylation in fused cells, leading to IFN-I production as an antiviral defense. However, SARS-CoV-2 encodes nonstructural and accessory proteins that antagonize host innate immunity. The viral main protease 3-chymotrypsin-like protease, which is highly conserved among coronaviruses, disrupts the assembly of STING signalosome complexes, impairing downstream signal transduction^82^^,^^83^. Moreover, SARS-CoV-2-induced mitochondrial damage also contributes to cGAS–STING activation. Notably, delayed IFN-I responses facilitate viral replication while inducing excessive inflammation, potentially culminating in cytokine storms, disseminated intravascular coagulation, or multi-organ failure^84^, ^85^, ^86^, ^87^.

HIVs, which emerged through multiple independent zoonotic transmissions of simian immunodeficiency viruses from non-human primates, also activate the cGAS–STING axis during infection^73^^,^^88^, ^89^, ^90^. HIV-1-induced cGAS–STING activation promotes IFN-I production, which upregulates antiviral effector proteins that restrict HIV replication. IFN-I also enhances antigen presentation, chemokine production by antigen-presenting cells, B cell antibody responses, and T cell effector functions, particularly supporting CD4^+^ T cell recovery. Therefore, fine-tuning early IFN-I responses may represent a promising therapeutic strategy to prevent or mitigate HIV infection and improve clinical outcomes^10^^,^^91^^,^^92^.

Beyond antiviral immunity, the cGAS–STING pathway is also implicated in antibacterial defense. In the gut microbiota, outer membrane vesicles released by gram-negative bacteria can carry bacterial DNA to distal host cells, triggering cGAS activation^93^. Additionally, cyclic dinucleotides (CDNs) derived from bacterial metabolism, such as cyclic di-GMP and cyclic di-AMP, can directly bind and activate STING, representing another mechanism of cGAS-independent antibacterial immune^94^.

## 3 Clinical research progress of the STING drug

In recent decades, scientists have been striving to identify more effective and durable therapies for cancer. Among the various cancer immunotherapies, STING agonists have emerged as a particularly promising approach, which harness the patient's immune system to combat systemic tumors^4^^,^^95^^,^^96^. A wide range of STING agonists, including CDN-based molecules and non-CDN small molecules, have entered clinical trials (Table 1). Among the CDN-based agents, ADU-S100, MK-1454, SB11285, GSK3745417, and IMSA101 are under early-phase clinical development for various solid tumors and hematologic malignancies^97^, ^98^, ^99^, ^100^, ^101^, ^102^, ^103^. These compounds are primarily administered *via* intratumoral or intravenous routes and aim to stimulate type I interferon production to enhance anti-tumor immunity.

**Table 1:** Representative STING agonists in the clinical development stage.

Agonist				NCT number	Ref.
Types	Drug candidates	Indication	Status		
CDN	ADU-S100	Head and neck cancer/solid tumors	Phase 2/1 (T)	NCTO3937141/NCT02675439	97,98
	MK-1454	Solid tumors or lymphomas/HNSCC	Phase 1/2 (C)	NCTO3010176/NCT04220866	97,98
	SB 11285	Solid tumors	Phase 1 (C)	NCTO40966381	101
	GSK37454171	Relapsed or refractory myeloid malignancies	Phase 1 (T)	NCT05424380	102
	IMSA101	Refractory malignancies/oligoprogressive solid tumor malignancies/oligometastatic NSCLC and RCC	Phase 2/2/2 (C/T/T)	NCT04020185/NCT05846659/NCT05846646	103
Non-CDN	E−7766	Advanced solid tumors or Lymphomas/NMIBC	Phase 1/1 (T)	NCTO4144140/NCTO41090921	104,105
	CRD3874-SI	AML/solid tumors	Phase 1/1 (R)	NCT06626633/NCT06021626	106,107
	MK-2118	Solid tumors or lymphomas	Phase 1 (T)	NCT03249792	108
Nanodrug	ExoSTING	Advanced/metastatic, recurrent, injectable solid tumors	Phase 1/2 (C)	NCT04592484	112
	cGAMP MPs	EAE/MS	Unknown	NCT05705986	113
Inhibitors					
Types	Drug candidates	Indication	Status	Trials number	
cGAS	VENT-03	SLE/RA	Phase 2/1 (C)	Not yet.	114
	IMSB301	SLE	Phase 1	ISRCTN 90049550	

Abbreviations: R, recruiting; C, completed; T, terminated; AML, acute myeloid leukemia; NMIBC, non-muscle-invasive bladder cancer; HNSCC, head and neck squamous cell carcinoma; NSCLC, non small cell lung cancer; RCC, renal cell aarcinoma; EAE, encephalomyelitis; MS, multiple sclerosis; SLE, systemic lupus erythematosus; RA, rheumatoid arthritis.

Beyond CDNs, non-CDN STING agonists such as E-7766, CRD3874-SI, and MK-2118 are also being evaluated in patients with advanced or refractory solid tumors, including lymphomas and non-small cell lung cancer^104^, ^105^, ^106^, ^107^, ^108^. Notably, IMSA101 has progressed to multiple Phase 2 trials, reflecting growing confidence in its potential clinical utility. Nevertheless, these agents are still in relatively early stages of development, and no STING agonist has yet received regulatory approval. Their therapeutic efficacy has been limited in some trials, however, and adverse effects remain a concern. These challenges are often attributed to poor pharmacokinetic stability and suboptimal uptake by immune cells^109^, ^110^, ^111^.

In addition to conventional small molecules, a limited number of STING agonists are being explored in nanoparticle-based formulations (Table 1). ExoSTING, for instance, represents a novel nanodrug candidate currently in a Phase 1/2 clinical trial for advanced or metastatic solid tumors^112^. Another candidate, cGAMP MPs, is being evaluated for autoimmune conditions such as experimental autoimmune encephalomyelitis and multiple sclerosis, although clinical progress remains limited^113^. Compared to traditional small-molecule STING agonists, nanodrug formulations may offer advantages such as enhanced immune cell targeting, reduced systemic toxicity, and improved pharmacokinetics.

Inhibition of the cGAS–STING pathway has emerged as a promising therapeutic strategy for autoimmune and autoinflammatory diseases, where aberrant STING signaling contributes to pathological type I interferon responses. To date, however, no STING inhibitors have entered clinical trials. Instead, clinical efforts have focused on targeting the upstream sensor cGAS. Two cGAS inhibitors, VENT-03 and IMSB301 (Table 1), have advanced into early-phase clinical development for the treatment of SLE and rheumatoid arthritis. VENT-03 has completed a Phase 1 study in healthy volunteers and is poised to enter Phase 2 trials^114^. IMSB301, meanwhile, is currently under evaluation in a Phase 2 trial for SLE (ISRCTN 90049550, ISRCTN.com). These clinical investigations represent important progress in translating innate immune modulation into novel therapies for interferonopathies. Further research is needed to develop potent and selective STING inhibitors with favorable pharmacokinetics and safety profiles to complement existing approaches targeting cGAS.

Although significant progress has been made in the clinical research of STING agonists, no STING-based therapies have yet been approved by regulatory authorities. Nanoparticle-based STING therapeutic strategies remain at an early stage of clinical development. Nevertheless, as the application of nanotechnology continues to expand, the development of nanoparticle delivery systems offers new possibilities for overcoming the major limitations of conventional small-molecule STING agonists, including high polarity, poor pharmacokinetics, and systemic toxicity, thus addressing key bottlenecks in their clinical application.

However, despite the promising potential of nanotechnology-supported STING formulations in overcoming these challenges, several obstacles persist in clinical translation. One of the most significant barriers is the stability of STING agonists, especially in long-term treatments, where environmental factors (*e*.*g*., pH and temperature) may lead to the loss of activity, thus compromising therapeutic efficacy. Furthermore, the size, surface chemistry, and drug-loading capacity of nanoparticles can affect their distribution and metabolism within the body, potentially triggering adverse reactions, particularly with prolonged use. Additionally, the immune activation induced by STING agonists may lead to immune-related adverse events, including autoimmune reactions, excessive inflammation, and organ damage, which could limit the broader clinical application of these therapies.

To address these challenges, numerous research groups are actively working on the development of STING nanoparticle platforms with enhanced biocompatibility, controlled stability, and improved delivery efficiency, aiming to reduce toxicity risks and enhance therapeutic outcomes. In the context of cancer immunotherapy, several innovative STING delivery strategies have been proposed, including co-delivery with metal ions (such as manganese) to enhance immune activation, the delivery of synthetic STING agonist mimetics, and the design of membrane-derived biomimetic vesicles^115^, ^116^, ^117^. These platforms demonstrate significant potential for overcoming existing delivery barriers and may accelerate the clinical translation of STING-based therapies in oncology. However, further clinical data and long-term safety assessments are required to ensure their widespread applicability.

## 4 Nanotechnology-driven STING regulation in cancer

### 4.1 Strategies for co-delivery of STING agonists and anticancer agents

Numerous studies have demonstrated the potential application of STING agonists in cancer therapy, and these molecules have emerged as promising targets for the development of anticancer drugs. STING agonists have shown significant therapeutic efficacy across various tumor models, making them an attractive option for cancer immunotherapy^118^^,^^119^. However, despite these advances, challenges remain in achieving effective systemic delivery of STING agonists.

**Figure 4:**
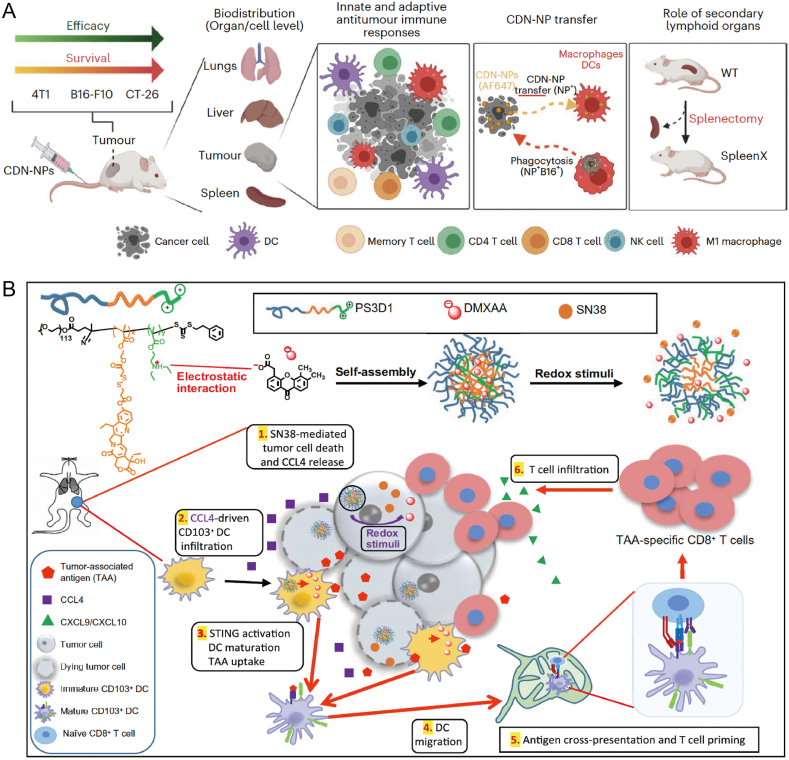
Strategies for co-delivery of STING agonists and anticancer agents. (A) The therapeutic efficacy of CDN-NPs was evaluated using various murine preclinical tumor models. Exploring the involvement of both cancer cells and host cells, with particular focus on immune cells, in determining the therapeutic outcomes of CDN-NPs^120^. Reprinted with the permission from Ref. 120. Copyright © 2023, Nat Nanotechnol. (B) Schematic illustration of the self-assembly of PS3D1@DMXAA NPs for redox-responsive drug release in tumor cells. The NPs induce tumor cell death and the secretion of chemokine CCL4, promoting the infiltration of CD103^+^ DCs into the TME. Concurrently, DMXAA activates the cGAS–STING pathway within CD103^+^ DCs. STING activation enhances the maturation of CD103^+^ DCs and facilitates the uptake of tumor-associated antigens (TAAs), promoting their migration to draining lymph nodes and the cross-presentation of TAAs. This process initiates the activation of TAA-specific effector CD8^+^ cytotoxic T lymphocytes. Furthermore, through the induction of CXCL9 and CXCL10, the immune-suppressive TME is reprogrammed to favor immune activation, supporting the recruitment of TAA-specific CD8^+^ T cells^123^. Reprinted with the permission from Ref. 123. Copyright © 2020, Sci Adv.

A study conducted by MIT reported the development of poly(*β*-aminoester)-based nanoparticles (NPs) covalently conjugated to CDNs, which serve as STING agonists. The conjugation was facilitated *via* a cathepsin-sensitive linker, allowing the NPs to be absorbed by target immune cells in both the tumor microenvironment and secondary lymphoid organs, such as the spleen. This approach not only eradicated tumors in murine models but also induced immune memory, preventing tumor recurrence (Fig. 4A)^120^. In another study, a multivalent STING agonist, a pH-sensitive polymer containing a seven-membered ring and tertiary amine (PC7A), was used to activate innate immune pathways. Unlike the natural STING ligand cGAMP, PC7A binds to a non-competitive STING surface site distinct from the cGAMP binding pocket, thus stimulating the production of pro-inflammatory cytokines. The polymeric nanoparticles exhibited slow and sustained immune activation compared to smaller molecular agonists, with effects detectable 12 h post-treatment and continued production of IFN-I at 48 h PC7A-induced immune responses were shown to depend on STING expression, CD8^+^ T cell activity, and antitumor responses, with synergistic therapeutic outcomes observed in subcutaneous tumor-bearing mice and resected human tumors and lymph nodes. The polymer-induced STING activation could provide new therapeutic opportunities for enhancing immune responses^121^. Additionally, ultrasound-driven activation of the cGAS–STING pathway has been explored for precise sonodynamic immunotherapy. In this approach, the research teams led by Zhen and Jiang designed a semiconducting polymeric nanoagonist (SPNM) capable of activating the cGAS–STING pathway *via* ultrasound. SPNM consists of a semiconducting polymeric photosensitizer coupled to the STING agonist MSA-2 through singlet oxygen-responsive linkers. Upon intravenous injection, SPNM accumulates in the tumor region through passive targeting. Under ultrasound stimulation, SPNM generates singlet oxygen, which disrupts the singlet oxygen-responsive group on the SPNM, releasing the STING agonist MSA-2. The released MSA-2 specifically binds to STING proteins within DCs in the tumor region, promoting the phosphorylation of TBK1 and IRF3, thereby stimulating the secretion of IFN-*β*. Simultaneously, the generated singlet oxygen induces immunogenic cell death (ICD) in tumor cells. These combined actions enhance dendritic cell maturation, increase the proliferation and infiltration of effector T cells, and strengthen antitumor immune responses, providing a targeted immunotherapy strategy for cGAS–STING pathway activation *via* ultrasound^122^.

Moreover, new strategies combining chemotherapy and immunotherapy have been developed. For example, triblock copolymer nanoparticles PS3D1@DMXAA enable the systematic co-delivery of the chemotherapy drug SN38 (7-ethyl-10-hydroxycamptothecin) and the STING agonist DMXAA (5,6-dimethylxanthenone-4-acetic acid) to tumors, aiming to modulate the tumor microenvironment (TME). The synergistic interaction between SN38 and STING activation was found to enhance antigen cross-presentation and convert the immune-suppressive TME into an immune-activated TME. PS3D1@DMXAA demonstrated robust therapeutic effects in three different murine tumor models and showed enhanced therapeutic efficacy when combined with anti-PD-1 treatment. This engineered nanoplatform offers a well-designed and effective immunotherapy combination strategy, turning “cold” tumors into “hot” tumors and addressing major challenges faced by immunotherapy (Fig. 4B)^123^.

These innovative nanotechnology-based strategies underscore the immense potential of STING agonists and their combination with other therapeutic modalities to improve cancer treatment outcomes.

### 4.2 Metal ion-based nanodelivery platforms for cGAS–STING pathway activation

Metal ions, such as manganese and zinc ions, have been reported to activate the cGAS–STING pathway, thereby enhancing immune therapy. An increasing number of studies are focusing on the fabrication of metal ion-loaded STING NPs to potentiate the STING-mediated antitumor immune response^124^, ^125^, ^126^, ^127^, ^128^.

**Figure 5:**
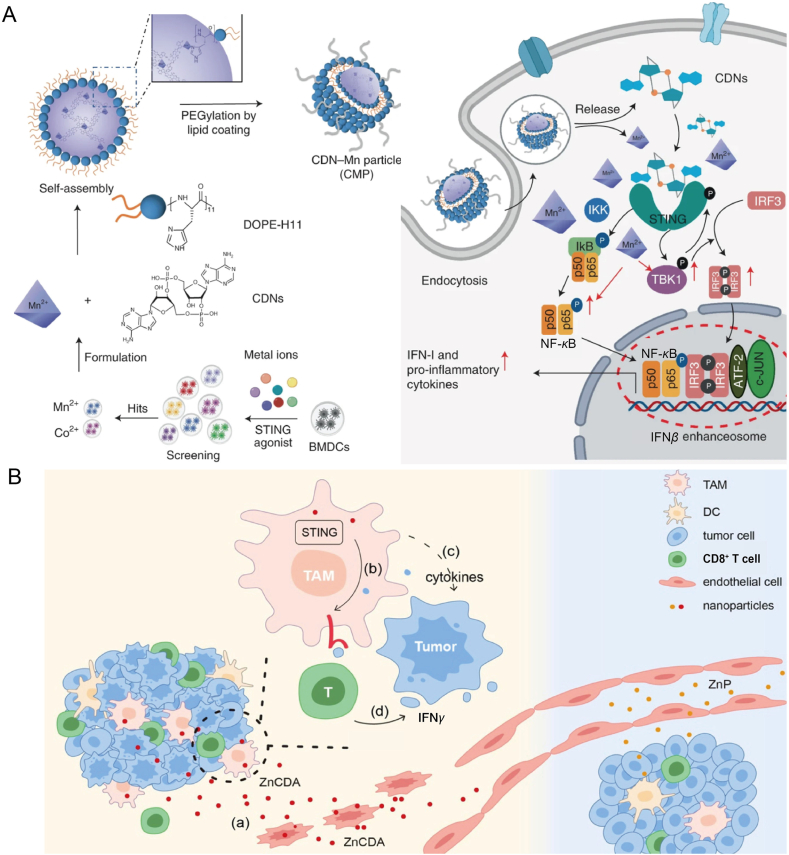
Metal ion-based nanodelivery platforms for cGAS–STING pathway activation. (A) The CMP is a composite material consisting of CDNs, Mn^2+^, phospholipid-(histidine)11 (DOPE-H11), and a PEG-lipid layer. Mn^2+^ enhances the IFN-I responses induced by STING agonists. Mn^2+^ and CDNs spontaneously self-assemble into a coordination polymer, with the CDN-Mn^2+^ complex being coated by DOPE-H11 *via* Mn-histidine interactions to form the CDN-Mn@DOPE structure. This is followed by PEGylation, resulting in the creation of the final CMPs^129^. Reprinted with the permission from Ref. 129. Copyright © 2021, Nat Nanotechnol. (B) Working model of ZnCDA in the TME: (a) Systemic administration of ZnCDA disrupts tumor vasculature by activating endothelial STING, thereby enhancing the accumulation of ZnCDA in the TME through an amplified EPR effect. (b) Once within the TME, ZnCDA selectively targets tumor-associated macrophages (TAMs) and modulates their antigen processing and presentation. (c) TAMs that internalize ZnCDA initiate an antitumor T cell response. (d) The antitumor efficacy of ZnCDA is independent of IFN*γ*, and preexisting T cells in tumors with T cell inflammation are sufficient to mediate the therapeutic effect^137^. Reprinted with the permission from Ref. 137. Copyright © 2022, Nat Nanotechnol.

James J. Moon's research team observed that the addition of manganese, a nutritional metal ion, to STING agonists resulted in a 77-fold enhancement of STING's antitumor efficacy compared to the use of STING agonists alone. Mn^2+^, in combination with CDN STING agonists, self-assembles into CDN-Mn^2+^ particles (CMPs), which, when administered locally or intravenously, trigger a robust antitumor immune response. This activation involved T cells, NK cells, and DCs, demonstrating significant therapeutic effects in multiple mouse tumor models using low doses of STING agonists (Fig. 5A)^129^. In another study, Yang's research team developed a manganese-phenolic network platform (TMPD) for the concurrent delivery of Mn^2+^ and doxorubicin (DOX), to amplify STING signaling and augment anticancer efficacy. TMPD effectively killed tumor cells by promoting cellular uptake and generating hydroxyl radicals (OH). DOX-mediated chemotherapy induced ICD in tumor cells, thereby triggering adaptive immune responses, while Mn^2+^ activated the cGAS–STING pathway, eliciting a strong innate immune response. Upon systemic injection, TMPD demonstrated excellent tumor suppression and a significant immune response. Additionally, by incorporating Mn^2+^ into TMPD, the platform enabled tumor-specific imaging, suggesting that TMPD is a promising nanoplatform capable of integrating imaging-guided cancer therapy with both innate and adaptive immune activation^130^. Furthermore, several studies have reported the use of manganese-containing STING NPs for tumor treatment^124^^,^^131^^,^^132^. Examples include Bi_2_−*x*Mn_*x*_O_3_ nanospheres for breast cancer therapy, pH-responsive biodegradable MnO@mSiO_2_-iRGD NPs for melanoma treatment, and *α*PDL1@MnO_2_ for inducing systemic antitumor immune responses and enhancing radiotherapy, and BPNS@Mn^2+^/CpG for effectively inhibiting tumor growth and eliciting robust immune memory. Additionally, Mn_3_O_4_@Au-dsDNA/DOX has been explored for melanoma treatment^128^^,^^133^, ^134^, ^135^, ^136^.

Besides manganese, zinc ions also show promise as metal ions for therapeutic purposes. Lin's research team^137^ developed a novel nanocoordination polymer composed of zinc phosphate and PEG-conjugated phospholipids, which can load both hydrophilic and hydrophobic components and be designed for stimulus-triggered drug release. This nanocoordination polymer features a non-toxic zinc phosphate core surrounded by a lipid layer, offering the advantage of controlled drug release, thereby increasing drug accumulation in tumors. Furthermore, a cyclic dinucleotide adenosine monophosphate (CDA), a nucleic acid molecule produced during host invasion by bacteria that potently activates the cGAS–STING pathway and innate immune responses, was encapsulated within this nanocoordination polymer to form ZnCDA. Intravenous injection of ZnCDA effectively prolonged CDA circulation in the body. Compared to traditional lipid nanoparticles delivering CDA, ZnCDA demonstrated advantages in preventing CDA degradation and prolonging blood circulation, offering superior antitumor effects compared to non-CDA STING agonists (Fig. 5B)^137^.

### 4.3 Design of nanocarriers for delivery of STING-activating mimetics

**Figure 6:**
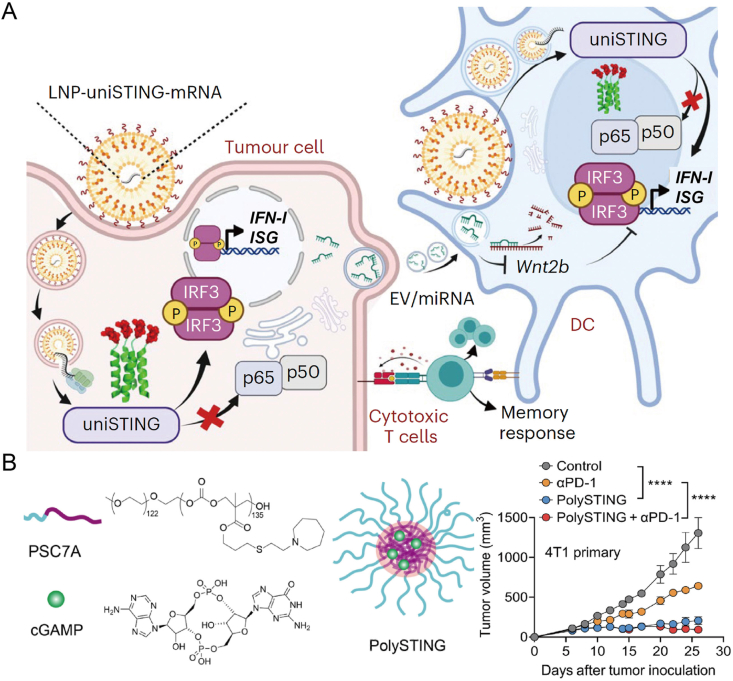
Design of nanocarriers for delivery of STING-activating mimetics. (A) LNP-uniSTING-mRNA induced continuous STING activation and facilitated EV-mediated communication between tumor cells and DCs. Upon LNP-uniSTING-mRNA treatment, both tumor cells and DCs expressed a universal STING mimic, which self-assembled into tetrameric units. These units subsequently formed a higher-order STING complex, leading to efficient downstream phosphorylation of IRF3, followed by the secretion of IFNs and ISG cytokines. EVs released from tumor cells treated with UniSTING further enhanced DCs functionality within the TME by secreting miRNAs, such as miR-130-3p, miR-15b-5p, and miR-16-3p, which specifically targeted Wnt2b and reduced immune-suppressive signaling molecules^138^. Reprinted with the permission from Ref. 138. Copyright © 2024, Nature Nanotechnology. (B) Schematic representation of PolySTING micelle nanoparticles showing dual STING activation through 2′,3′-cGAMP and PSC7A polymer. 2′,3′-cGAMP is encapsulated in micelles self-assembled from pH-sensitive PSC7A polymers. PSC7A has a significant therapeutic effect on mouse tumor model^139^. Reprinted with the permission from Ref. 139. Copyright © 2024, Sci Immunol.

UniSTING, a genetically engineered fusion of the highly stable tetramerization motif and the non-membrane binding domain of STING, can self-assemble into tetrameric STING structures, which further nucleate into polymerized STING independent of the endoplasmic membrane system. This design mimics the endogenous STING oligomerization induced by cGAMP, selectively activating the IRF3/IFN-I cascade. As a result, this engineered construct induces the activation of DCs in mice. Moreover, tumor cells treated with UniSTING release extracellular vesicles (EVs), which, through the secretion of miRNAs, enhance the functionality of DCs within the TME. These miRNAs specifically target Wnt2b and downregulate immune suppressive signaling molecules. Experimental validation demonstrated that LNP-uniSTING-mRNA therapy can elicit potent antitumor effects in various cancer models, including triple-negative breast cancer, lung cancer, melanoma, and both primary and metastatic liver malignancies (Fig. 6A)^138^. Furthermore, the degradable ultra-pH-sensitive (dUPS) polymer, PSC7A, serves as a vaccine adjuvant while maintaining its inherent pH sensitivity, antigen presentation capacity, and antitumor immune response. The degradability of dUPS also effectively reduces the toxicity of PSC7A. Notably, the combination of PolySTING with anti-PD-1 therapy achieves significant tumor growth suppression and enhances therapeutic efficacy across a broader range of treatment areas (Fig. 6B)^139^.

This therapeutic strategy offers a multifaceted approach to cancer treatment. By selectively activating the cGAS–STING pathway and utilizing targeted drug delivery systems such as LNP-uniSTING-mRNA and PSC7A, this approach circumvents the limitations of traditional therapies. It presents a unique opportunity to potentiate both innate and adaptive immune responses, potentially shifting the therapeutic paradigm for cancers that are resistant to conventional treatments. Additionally, the combination with checkpoint inhibitors, such as anti-PD-1, exemplifies how STING activation can be synergistically integrated with immunotherapy to enhance the body's antitumor response, leading to more effective and durable treatment outcomes. These advances indicate the growing potential of STING-based therapies in immuno-oncology and highlight the promise of integrating novel nanomaterials and immunomodulatory strategies in the fight against cancer.

### 4.4 Bionic nanocarriers for enhanced STING-mediated tumor immunotherapy

**Figure 7:**
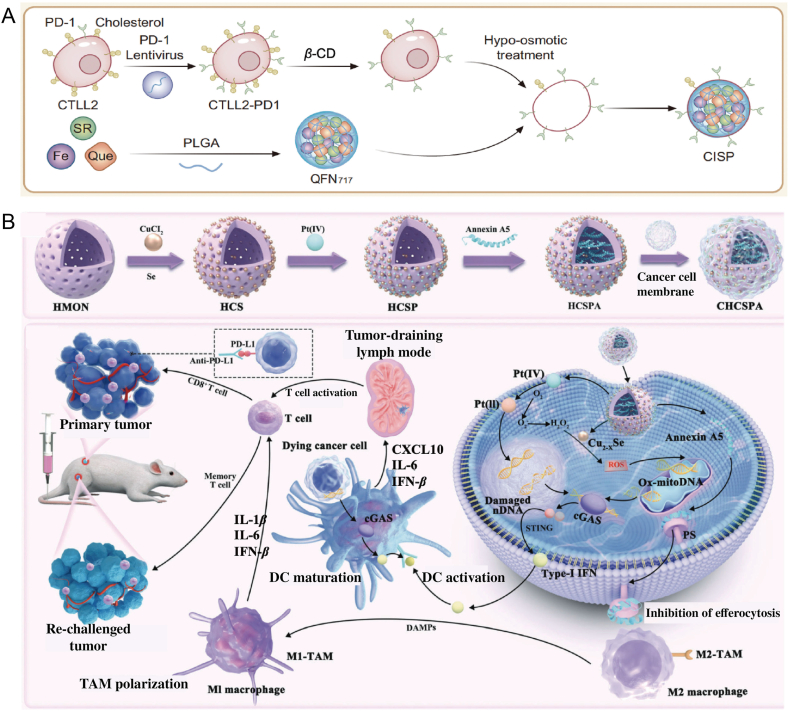
Biomimetic nanomaterials for enhanced STING-mediated tumor immunotherapy. (A) Schematic representation of the preparation process of CISP, highlighting its enhancement of tumor-targeted SR-717 delivery^143^. Reprinted with the permission from Ref. 143. Copyright © 2023, Nature Communications. (B) Synthesis of CHCSPA nanoparticles and the proposed innate immunotherapy strategy for inhibiting primary and rechallenged tumors through the release of damaged dsDNA and activation of the cGAS–STING pathway. The combination of chemotherapy and chemodynamic therapy induced by CS and Pt (IV) simultaneously damages nuclear and mitochondrial DNA, subsequently promoting DCs maturation and M1 polarization of tumor-associated macrophages *via* the activation of the cGAS–STING pathway. Furthermore, the released Annexin A5 protein prevents the exposure of phosphatidylserine on dying tumor cells, inhibiting efferocytosis and enhancing the release of dsDNA *via* secondary necrosis^145^. Reprinted with the permission from Ref. 145. Copyright © 2023, Small.

Cell membrane-coated nanoparticles have emerged as an important biomimetic drug delivery system, attracting considerable research interest in recent years^140^, ^141^, ^142^. They exhibit numerous advantages in targeted drug delivery. However, the size, morphology, and protein composition of the cell membranes used in these delivery systems differ from those of normal blood cells, thus making it challenging to avoid being recognized as foreign entities and subsequently phagocytosed by immune cells. As such, there is an urgent need for alternative strategies in biomembrane engineering to enhance their resistance to clearance and improve their bioactivity. Liposomes, which share a similar basic surface structure with biological membranes, have garnered significant attention. Researchers have noted that increasing the cholesterol content in liposomes accelerates their clearance from the bloodstream, although the underlying mechanism remains unclear. However, it is worth investigating whether reducing cholesterol content in biological membranes could enhance their performance. *β*-cyclodextrin, a biocompatible compound, has been reported to be capable of removing cholesterol. Researchers have utilized *β*-cyclodextrin-treated membranes derived from T cells overexpressing PD-1 to encapsulate SR-717 (a cAMP analog) loaded on quercetin iron nanoparticles. This system was termed CISP, referring to low-cholesterol membrane-encapsulated immune checkpoint blockers, STING agonists, and photothermal agents. The membranes with reduced cholesterol content exhibited a 50% reduction in the uptake of CISP by monocytes in the blood, while retaining their tumor-targeting ability. Consequently, CISP effectively delivered SR-717 to tumor sites to activate the local cGAS–STING pathway, while the PD-1 blockade on CISP surfaces inhibited PD-L1 expression in tumor cells (Fig. 7A)^143^.

To maximize the therapeutic potential of cancer cell membranes, sonodynamic therapy, and Mn^2+^ in cancer treatment, a research team led by Professors Mingwu Shen and Xiangyang Shi at Donghua University synthesized AM-biomimetic camouflage nanogels (PVCL NGs) encapsulating MnO_2_ nanoparticles using a simple method. These nanogels were designed to load the sonosensitizer Ce6 and the immune adjuvant cGAMP. The resulting NG-based therapeutic nanovaccine serves a dual purpose: it can directly activate immune cells while also directly killing tumor cells and inducing ICD, thereby achieving effective tumor prevention and direct treatment *via* full-cycle immune modulation. The team first synthesized pH/ROS-responsive PVCL NGs using a boronate ester-based crosslinker *via* precipitation polymerization. Then, MnO_2_ nanoparticles were synthesized *in situ* inside the nanogels; subsequently, Ce6 was loaded *via* Mn-N coordination, and cGAMP was adsorbed electrostatically; finally, AM was coated on the surface to form PMCG@AM NGs^144^.

Studies have demonstrated that dying tumor cells release dsDNA, which can activate the cGAS–STING signaling pathway. However, due to phagocytosis, dying tumor cells are engulfed and cleared before the damaged dsDNA can be released, leading to immune tolerance and immune escape. To address this, cancer cell membrane-mimicking nanocomposites with tumor immunotherapy effects were synthesized by enhancing the cGAS–STING pathway and inhibiting phagocytosis. Upon internalization by cancer cells, these nanocomposites trigger combined chemotherapy/chemokinetic therapy, damaging both nuclear and mitochondrial DNA. Additionally, the release of Annexin A5 protein inhibits phagocytosis by blocking phosphatidylserine exposure, promoting immune-stimulatory secondary necrosis and the release of dsDNA. These dsDNA fragments act as molecular patterns of immunogenic damage, escaping from cancer cells, activating the cGAS–STING pathway, enhancing cross-presentation within dendritic cells, and promoting M1 polarization of tumor-associated macrophages. *In vivo* experiments demonstrated that the proposed nanocomposites effectively recruit cytotoxic T cells and promote long-term immune memory. Furthermore, when combined with immune checkpoint blockade, they enhance immune responses. Therefore, this novel biomimetic nanocomposite presents a promising strategy for eliciting adaptive anti-tumor immune responses (Fig. 7B)^145^.

This innovative approach not only improves the efficacy of cancer immunotherapy but also offers insights into overcoming key challenges such as immune evasion and phagocytic clearance, which have long hindered the success of nanoparticle-based treatments in clinical applications.

## 5 Nanotechnology-driven STING regulation in inflammation and autoimmune diseases

### 5.1 Nanotechnology-driven STING regulation in inflammatory diseases

Inhibiting the cGAS–STING signaling pathway has emerged as a promising therapeutic strategy for treating inflammatory diseases. Overactivation of STING contributes to chronic inflammation by driving excessive production of pro-inflammatory cytokines, which can exacerbate tissue damage. By selectively targeting and suppressing STING activity, it is possible to modulate the immune response, reduce inflammation, and promote tissue healing. Recent advancements in nanotechnology, including the use of lipid nanocapsules and microneedle systems, allow for precise delivery of STING inhibitors, offering a controlled and targeted approach with reduced side effects. This targeted inhibition of STING presents a novel and effective strategy for managing immune-mediated diseases.

**Figure 8:**
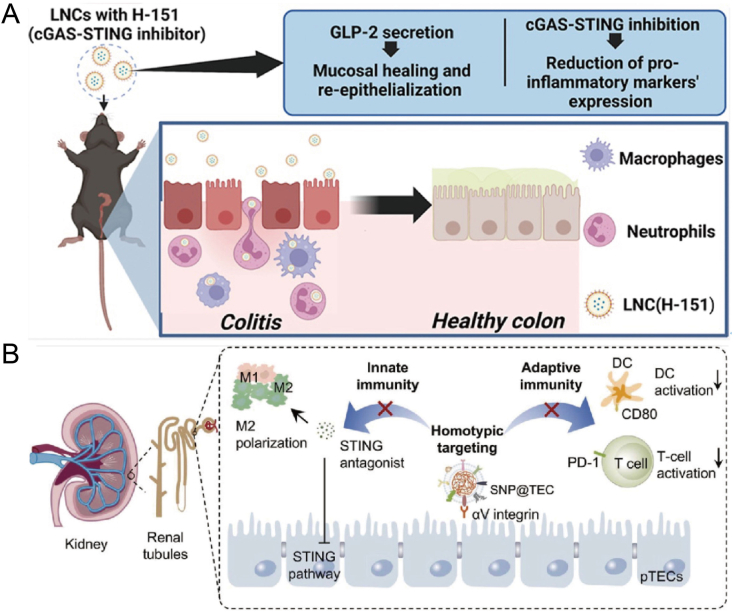
Nanotechnology-driven STING regulation in inflammatory diseases. (A) The STING inhibitor H-151 was encapsulated in LNCs to form H-151, which induced GLP-2 secretion while selectively targeting the cGAS–STING pathway and its downstream markers (TBK1 and pTBK1). This formulation also reduced pro-inflammatory cytokine levels (TNF-*α* and CXCL10) in mouse macrophages^146^. Reprinted with the permission from Ref. 146. Copyright © 2025, Mol Pharm. (B) Bionic SNP@TEC enhances AKI treatment by coordinating both innate and adaptive immune responses. It targets renal tubular cells *via* homologous targeting, delivering antagonists that suppress inflammation caused by STING activation. Additionally, SNP@TEC, through its cell membrane PD-L1, blocks CD80 on dendritic cells, inhibiting T cell activation and promoting anti-inflammatory adaptive immunity^147^. Reprinted with the permission from Ref. 147. Copyright © 2024, Nano Today.

Targeting the cGAS–STING signaling pathway has emerged as a promising strategy for developing novel oral therapies for IBD. In this study, different STING inhibitors were screened in mouse macrophages *in vitro*. The STING inhibitor H-151 was then encapsulated in lipid nanocapsules (LNCs), which, due to their inherent ability to induce the secretion of glucagon-like peptide 2 (GLP-2) (a regenerative peptide), were assessed for their therapeutic potential. The study demonstrated that H-151-loaded LNCs could selectively target the cGAS–STING pathway and its downstream key markers (including TBK1 and pTBK1), while simultaneously reducing the expression of pro-inflammatory cytokines (such as TNF-*α* and CXCL10) in mouse macrophages. In an *in vivo* acute dextran sulfate sodium-induced colitis mouse model, oral administration of H-151 significantly reduced pro-inflammatory cytokines to levels comparable to the control healthy group, while promoting mucosal healing. This scalable and cost-effective nanodrug demonstrates promising therapeutic potential as an alternative oral treatment for IBD and warrants further investigation for its clinical application (Fig. 8A)^146^.

Acute kidney injury (AKI) and other inflammatory diseases are challenging due to their complex mechanisms. AKI is often triggered by infections, sepsis, surgeries, shock, or nephrotoxic drugs. Small molecule inhibitors like C176, which target the cGAS–STING pathway, show promise in reducing AKI symptoms by modulating inflammation. However, their clinical use is limited by *in vivo* instability, short half-life, and poor renal targeting. To overcome these issues specifically, biomimetic nanoparticles derived from cell membranes, particularly renal tubular epithelial cells, enhance renal targeting *via* homologous targeting. This method also leverages PD-L1 expression on the membrane to modulate immune responses, such as inhibiting dendritic cell activation or promoting regulatory T cell differentiation, reducing inflammation. This strategy improves STING inhibitor delivery to the kidneys and reduces systemic inflammation, providing a more effective targeted therapy for AKI (Fig. 8B)^147^.

Excessive activation of the cGAS–STING pathway leads to insulin resistance and inflammation in type 2 diabetes. Nanomaterials can effectively regulate this pathway by targeted delivery of STING inhibitors, alleviating insulin resistance and improving glucose metabolism^148^. Additionally, studies have shown that abnormal activation of the cGAS–STING pathway is a key mechanism in immune-mediated kidney damage and neuroinflammation. By nanotechnology-driven STING regulation, the cGAS–STING pathway can be precisely modulated to reduce excessive inflammation, protect neuronal cells, and improve cognitive function^149^^,^^150^.

### 5.2 Nanotechnology-driven STING regulation in autoimmune diseases

**Figure 9:**
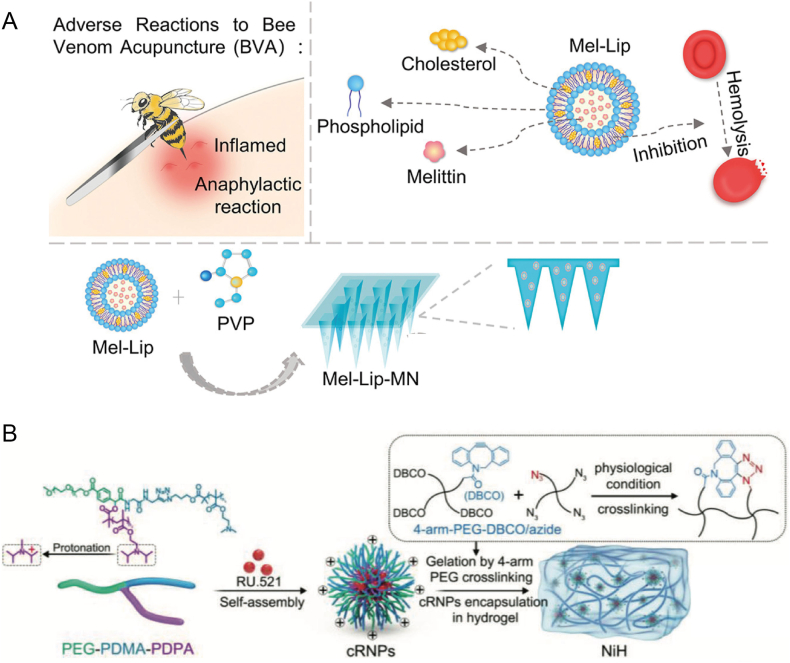
Nanotechnology-driven STING regulation in autoimmune diseases. (A) BVA encapsulated in liposomes is formulated into soluble microneedles loaded with bee Mel-Lip for transdermal delivery in the treatment of RA^151^. Reprinted with the permission from Ref. 151. Copyright © 2025, Nanomedicine. (B) NiH co-delivered cfDNA-scavenging cNPs and cGAS inhibitors to draining LNs for systemic immunosuppression in RA therapy. The NiH system consists of an injectable hydrogel encapsulating cRNPs, formed by crosslinking 4-arm-PEG-DBCO with 4-arm-PEG-N3 using copper-free click chemistry. The cRNPs were incorporated into the hydrogel prior to crosslinking^152^. Reprinted with the permission from Ref. 152. Copyright © 2023, Adv Sci (Weinh).

Bee venom acupuncture (BVA) has shown significant potential in the treatment of rheumatoid arthritis; however, it faces challenges related to the pain and allergic reactions associated with bee stings. A key hurdle in utilizing bee venom is the instability of melittin, the primary anti-inflammatory component of bee venom, which rapidly degrades upon oral administration, leading to reduced efficacy and increased toxicity. This study proposes encapsulating melittin within liposomes to enhance its stability, minimize its side effects, and broaden its clinical applicability. Furthermore, advancements in microneedle technology, which target the stratum corneum and bypass gastrointestinal concerns, offer a novel approach for RA treatment. By utilizing soluble microneedles loaded with melittin-encapsulated liposomes (Mel-Lip), effective transdermal delivery was achieved. Results from an adjuvant-induced RA animal model demonstrated that Mel-Lip microneedles significantly improved foot health, repaired cartilage, and reduced inflammation markers, underscoring the potential of transdermal nanocarrier microneedles as a patient-friendly therapeutic approach for RA (Fig. 9A)^151^.

Another study reported the development of cationic nanoparticles (cNPs) loaded with a cGAS inhibitor, RU.521, to create a nanodrug, cRNP, for RA treatment. The cRNP nanodrug was then incorporated into a hydrogel to form a nanoparticle-hydrogel (NiH) system, prolonging the release of cRNPs and RU.521. The DIR dye was used as a model fluorescence probe to evaluate the drug retention capability of DIR-loaded cNPs-in-water gel (DIR/cNPs-H).

Studies were performed on mice receiving tail-base injections of DIR/cNPs-H or control formulations; results showed a significant increase in DIR accumulation in draining lymph nodes over 1–5 days compared with free DIR. Specifically, compared to DIR/cNPs, the DIR/cNPs-H formulation prolonged DIR retention in the lymph nodes (LNs), with retention on Day 5 being 1.47 times and 7.8 times higher than DIR/cNPs and free DIR, respectively. On Day 5, the fluorescence intensity of DIR/cNPs-H in the LNs was 217% higher than that of DIR/cNPs, demonstrating effective drug delivery and retention in LNs, while also reducing systemic drug spread. *Ex vivo* imaging of excised LNs confirmed effective DIR retention in LNs treated with DIR/cNPs-H (Fig. 9B)^152^.

In conclusion, these studies underline the growing potential of advanced nanocarrier systems, including liposomes, microneedles, and hydrogel formulations, for delivering bioactive agents such as melittin and STING inhibitors in a controlled and targeted manner. The integration of these nanotechnologies into therapeutic strategies for complex diseases like rheumatoid arthritis and inflammatory bowel disease opens new avenues for more effective, less invasive, and patient-friendly treatments. Future research should focus on optimizing the pharmacokinetic profiles and therapeutic efficacy of these formulations, as well as exploring their use in combination therapies to further enhance their clinical outcomes.

## 6 Nanotechnology-driven STING regulation in infectious diseases

**Figure 10:**
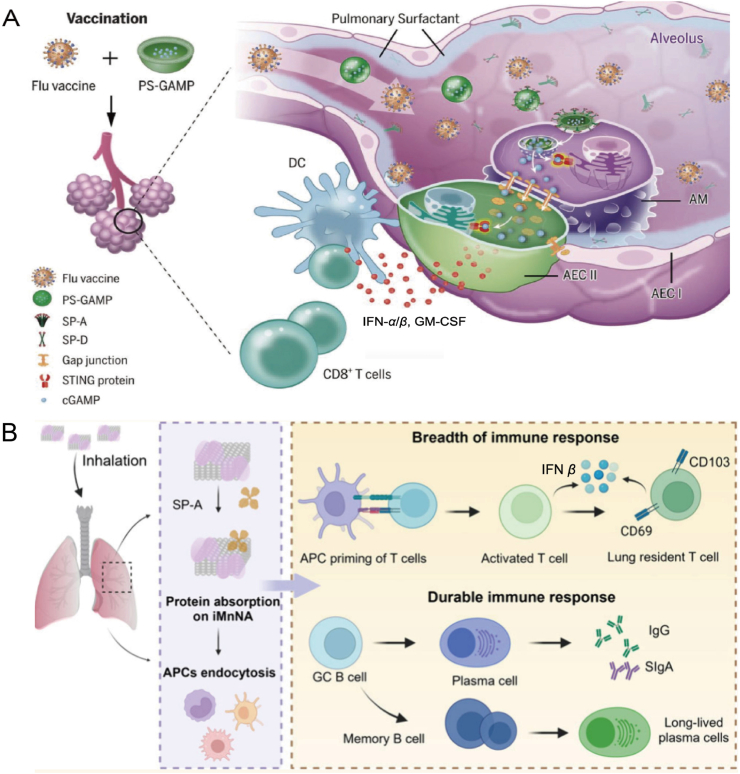
Nanotechnology-driven STING regulation in infectious diseases. (A) In the alveoli, SP-A or SP-D enters AMs through SP-A- or SP-D-mediated endocytosis. Once inside, cGAMP is released into the cytosol and diffuses into AECs *via* gap junctions. There, it activates the cGAS–STING pathway, triggering a strong production of type I immune mediators. These mediators enhance the recruitment and differentiation of CD11b^+^ DCs, which drive robust antiviral responses, including activation of CD8^+^ T cells and humoral immunity^153^. Reprinted with the permission from Ref. 153. Copyright © 2020, Science. (B) iMnNA-formulated candidate MnVac was used for pulmonary immunization. By binding to albumin, iMnNA promotes the formation of an *in situ* local PS protein corona, which enhances the accumulation of the vaccine in the lung parenchyma and facilitates the uptake of antigens by antigen-presenting cells^157^. Reprinted with the permission from Ref. 157. Copyright © 2025, ACS Nano.

In the context of modern immunotherapy, the cGAS–STING pathway has attracted substantial attention due to its pivotal role in innate immune responses to various infections, including viral and bacterial pathogens. A novel pulmonary biomimetic nanoparticle (PS-GAMP) was designed based on cGAMP to simulate influenza virus-induced lung infections. The study found that PS-GAMP could be released into the cytoplasm without disrupting pulmonary surfactant or alveolar epithelial cell (AEC) barriers. The cGAMP released from PS-GAMP entered AECs *via* gap junctions, activating the cGAS–STING signaling pathway within these cells. This activation induced a robust type I immune response, stimulating alveolar macrophages (AMs) and AECs to orchestrate robust humoral immunity and CD8^+^ T cell-mediated protective immune responses. This immune activation provided robust protection against various strains of influenza viruses, demonstrating the therapeutic potential of PS-GAMP for combating respiratory infections. This nanoparticle-based approach thus offers a promising alternative to traditional vaccination strategies, specifically by enhancing local immunity in the lungs and providing broader protection against viral mutations (Fig. 10A)^153^^,^^154^.

The development of effective vaccine adjuvants, especially those targeting viral infections like SARS-CoV-2, has become a crucial focus in current immunotherapeutic research. Recent studies have explored the combination of Toll-like receptor 9 (TLR9) agonists and STING agonists as adjuvants to enhance immune responses. Specifically, CpG-2722, a TLR9 agonist, was combined with various CDNs, including 2′3′-c-di-AM(PS)2, and demonstrated promising results in immunized mice, notably enhancing humoral immune responses and activating both B and T cell responses. These agonists effectively promoted germinal center B cell reactions, leading to stronger antibody production and more robust protection against SARS-CoV-2. This finding underscores the substantial potential of combining TLR9 and STING agonists to enhance vaccine efficacy, providing a novel strategy for the development of next-generation immunotherapies^155^^,^^156^.

In another study, an inhalable Mn nanoparticle adjuvant (iMnNA) was used to formulate a candidate mucosal vaccine (MnVac), which effectively promoted vaccine accumulation in the lungs, enhanced antigen presentation, and induced sustained antibody responses. When combined with the SARS-CoV-2 receptor binding domain dimer, MnVac significantly boosted antibody responses even at low doses, by activating the cGAS–STING pathway and promoting both systemic and local immune responses (Fig. 10B)^157^. Additionally, a bacterial cell nanoparticle (CNP) vaccine, named Pa-STING CNP, was developed, containing an adjuvant core that activates the cGAS–STING pathway. This nanoparticle platform showed promising potential for providing effective immune protection against multidrug-resistant pathogens such as *Pseudomonas aeruginosa*^158^.

In conclusion, these studies highlight the promising potential of combining TLR9 and STING agonists with nanoparticle technology to significantly enhance vaccine efficacy, prolong immune responses, and provide novel strategies for combating a range of viruses and antibiotic-resistant pathogens. Specifically, these innovative vaccine platforms and adjuvants represent valuable approaches for the future development of vaccines targeting respiratory viruses like SARS-CoV-2. However, further clinical validation and safety assessments are required to ensure the effectiveness and feasibility of these technologies in real-world applications.

## 7 Challenges and limitations

The application of nanomaterials in the regulation of the cGAS–STING signaling pathway holds considerable promise, particularly in the field of immunotherapy. However, several technical challenges must be addressed before these systems can be translated into clinical practice.

(1) Biological safety and immunogenicity of nanomaterials. The activation of the cGAS–STING signaling pathway is crucial in immunotherapy, but the use of nanomaterials to modulate this pathway can trigger potent immune responses. Nanocarriers designed with biomimetic membranes and complex structures may be recognized as foreign by the host immune system, leading to immune activation, cytokine storms, systemic inflammation, off-target effects, or immune tolerance. These issues represent some of the most significant challenges facing the clinical translation of nanomaterials that regulate the cGAS–STING pathway. To mitigate these risks, several strategies have been proposed in current research: optimizing the surface characteristics of nanomaterials (*e.g.*, through PEGylation or biomimetic design) to reduce immune recognition and immunogenicity^159^^,^^160^; employing immunomodulatory agents and biodegradable materials to minimize cytokine storms, inflammation, and long-term toxicity^161^. Long-term studies on the immunogenicity and biodegradability of nanomaterials are essential to enhance their biosafety and ensure their successful clinical application.
(2) Targeting specificity and biodistribution. Effective modulation of the cGAS–STING pathway relies on the activation of the immune system, making the targeted delivery of nanomaterials to specific tumors or inflamed regions critical. However, biological barriers in the tumor microenvironment, such as the vascular endothelial barrier and the lymphatic system, along with tumor heterogeneity, often impede the efficient penetration and accumulation of nanomaterials, thereby reducing therapeutic efficacy. Accordingly, nanocarriers designed to modulate STING must be optimized for both targeting specificity and biodistribution to ensure effective immune activation in desired regions.
(3) Maintaining membrane integrity in biomimetic nanomaterial preparation. Nanomaterials, particularly biomimetic nanocarriers, require the preservation of membrane integrity and functionality to effectively regulate the cGAS–STING pathway. However, the inherent complexity of cell membranes and membrane proteins poses significant challenges in maintaining membrane stability during preparation. These membranes are prone to damage during synthesis, storage, or use, which may compromise their biocompatibility and targeting ability.
(4) Scale-up, quality control, and storage. For the clinical application of STING-modulating nanotherapies, large-scale production, quality control, and storage of nanomaterials are critical issues. During manufacturing, it is essential to ensure the consistency and stability of the nanomaterials, which is pivotal for their clinical effectiveness. Additionally, optimizing storage conditions to preserve the efficacy and safety of these materials over time is crucial^162^^,^^163^.

Addressing these multifaceted technical challenges requires a multidisciplinary approach involving materials science, immunology, bioengineering, and regulatory science. Such collaboration is essential to ensure that nanomaterials designed to regulate the cGAS–STING signaling pathway can be safely and effectively translated into clinical applications.

## 8 Conclusions and perspectives

With the ongoing advancement of nanotechnology and the regulation of the cGAS–STING pathway, nanotechnology-based strategies targeting STING activation or inhibition are expected to play an increasingly significant role in precision therapy, personalized medicine, and the treatment of various immune-related diseases in the future.

In oncology, optimizing nanodelivery systems and enhancing their targeting capabilities offer the potential to combine STING agonists with existing therapies, such as immune checkpoint inhibitors, for stronger synergy and improved cancer immunotherapy. This synergistic combination strategy is especially important for tumors with strong immune tolerance, where precise STING activation could overcome current immunotherapy limitations, driving innovation. In infectious diseases, integrating nanotechnology-driven STING activation strategies promises more efficient antiviral therapies, especially for viruses like COVID-19 and HIV, which have rapid mutation rates and immune evasion mechanisms. By activating STING, these strategies can boost the host's immune response, providing new avenues to combat these infections. By contrast, for autoimmune and inflammatory diseases, research should focus on precise STING inhibition to avoid excessive immune suppression. Nanodelivery systems combined with immune-modulatory strategies could effectively reduce inflammation while preserving immune function, offering better management options for these conditions. Additionally, the cGAS–STING pathway is critical in metabolic diseases and aging, where its activation is linked to chronic inflammation driving metabolic dysfunction. Modulating STING could alleviate inflammation in metabolic disorders and potentially delay age-related immune decline.

Moreover, the integration of artificial intelligence (AI) and machine learning into drug design, disease prediction, and personalized treatment plans offers exciting opportunities for optimizing nanotechnology-based STING modulation strategies. AI-driven insights can enhance drug delivery optimization and enable more accurate patient data analysis, facilitating the integration of nanomedicine with precision medicine and offering more scientifically grounded and effective solutions for treating immune-related diseases.

In conclusion, nanotechnology-based cGAS–STING pathway activation and inhibition strategies show unprecedented potential for the treatment of various diseases. As research continues and technology advances, these nanotechnology-driven immune modulation approaches are poised to play a pivotal role in the treatment of cancer, infectious diseases, and immune system dysregulation, opening the door to more precise and personalized therapeutic interventions.

## Author contributions

Qianwen Mu and Qihang Huang contributed equally to this work and jointly drafted the manuscript. Haolan Deng provided critical revisions and editorial input. Gang Liu and Chao Liu offered valuable suggestions and contributed to critical revisions of the manuscript. This manuscript represents the collective effort of all authors, and all authors have approved the final version.

## Conflicts of interest

The authors declare no conflicts of interest.
